# Marginal Bone Loss around Implants with Internal Hexagonal and Internal Conical Connections: A 12-Month Randomized Pilot Study

**DOI:** 10.3390/jcm10225427

**Published:** 2021-11-20

**Authors:** Pablo Galindo-Moreno, Ada Concha-Jeronimo, Lucia Lopez-Chaichio, Roque Rodriguez-Alvarez, Elena Sanchez-Fernandez, Miguel Padial-Molina

**Affiliations:** 1 Department of Oral Surgery and Implant Dentistry, School of Dentistry, University of Granada, 18071 Granada, Spain; elenasf@ugr.es (E.S.-F.); mipadial@ugr.es (M.P.-M.); 2 Research Group in Oral Biology and Regeneration (CTS-1028), Junta de Andalucia, 18071 Granada, Spain; roquejesusra@correo.ugr.es; 3 PhD Program in Clinical Medicine and Public Health, University of Granada, 18016 Granada, Spain; adaconcha@correo.ugr.es (A.C.-J.); luciaxa@correo.ugr.es (L.L.-C.)

**Keywords:** marginal bone loss, implant–prosthesis connection, peri-implantitis, dental implants

## Abstract

The aim of this study was to analyze the differences in terms of the marginal bone level (MBL) around implants with either an internal conical or an internal hexagonal implant–prosthesis connection. A randomized clinical trial included patients in need of a single implant-supported restoration. The implant–prosthesis connection was either internal conical or internal hexagonal while maintaining the same type of implant macro- and microarchitecture. Clinical and radiographical variables were registered up to 12 months of follow-up, including MBL. A total of 30 patients were included in the study. The main outcome variable, MBL 12 months after prosthesis delivery, was statistically different in both groups: −0.25 (0.12) vs. −0.70 (0.43) (conical vs. hexagonal; *p* = 0.033). Differences were also observed at the 3- and 6-month follow-up visits as well as for the MBL change from prosthesis delivery to the 12-month follow-up (−0.15 (0.13) vs. −0.56 (0.44); conical vs. hexagonal; *p* = 0.023). Correlations between MBL around the implants and radiographic measurements on the adjacent teeth, buccal bone to implant, tissue thickness or keratinized tissue were not significant neither globally nor when analyzed independently by group. In view of such results, it can be concluded that single-unit restorations with internal hexagonal-connection implants induce higher marginal bone loss after 12 months of follow-up from prosthesis delivery than internal conical-connection implants.

## 1. Introduction

Long-term clinical success in implantology is conditioned by several factors. The red line between health and pathology in implant-supported prosthetic treatments is defined by the progression of marginal bone loss (MBL) in the bone surrounding the implant neck [1]. In this context, the biological transition between soft and hard tissues and the restorative margin between the implant and the prosthetic elements are capital for bone maintenance. It is commonly known that the type of implant-to-crown connection is one of the keys to the response of biological components.

An International Expert Meeting held in Rome in 2013 and sponsored by the Camlog Foundation stated that crestal bone remodeling is observed for both external and internal connections regardless of whether they are conical or butt-joint [2]. However, although MBL can be observed around every type of implant–prosthesis connections, there are large differences between them. This is because the implant–prosthesis connection can be analyzed from different points of view: (1) vertical position with respect to the surrounding tissues, distinguishing between tissue-level and bone-level implants; (2) horizontal distance between the prosthesis and the implant’s outer dimension at the level of the connection, which defines platform-switching connections or butt-joint connections; (3) abutment fitting in relation to the implant, classified as external (flat or hexagonal) or internal (Morse, conical, hexagonal, octagonal, trilobed, etc.) connections. These different features lead to important differences in the effect of load distribution from the prosthesis to the implant and, consequently, from the implant to the surrounding bone [3]. In addition, micromovements of the prosthesis at the connection with the implant could also allow microbiological contamination and inflammation that would affect the surrounding bone [4].

Few systematic reviews or clinical studies have found no significant differences between internal conical-connection implants and external hexagonal-connection implants in terms of MBL or survival rates [5,6]. Other meta-analyses support the opposite: internal-connection implants have lower MBL compared to external connections implants [7,8]. This is confirmed in clinical studies [9,10], even irrespectively of bone type and nature [11,12]. These studies do not consider the influence of other important aspects beside the prosthetic connection that may influence MBL, such as implant surface characteristics, implant’s micro- and macroarchitecture, thread design, etc. Thus, the results from those comparison studies could be distorted [13]. In turn, Peñarrocha-Diago et al. demonstrated that in implants with similar macroarchitecture and surface features but different neck design and prosthetic connection, external-connection implants showed a higher MBL in comparison with internal-connection implants, irrespectively of implant location [14]. Consequently, in summary, implants with the internal connection are widely recommended over implants with the external connection.

Different internal connections are also available. In this sense, Schmitt et al. indicated that a conical implant–prosthesis connection seems to produce less MBL in vivo in comparison with nonconical connection systems [13]. Similarly, Laurell and Lundgren also found statistically significant differences in terms of MBL after 5 years of operation, being lower in implants with internal conical connections [15]. Comparable findings have been reported more recently by other authors [16]. However, we have only found two studies that specifically evaluated differences between internal conical and internal hexagonal implant–prosthesis connections [17,18]. One of them included implants from different manufacturers [18] while the other [17] did not find statistically significant differences. Thus, more information is clearly needed on this topic.

So, the aim of this study was to analyze differences in terms of MBL around implants with similar macroarchitecture and surface characteristics but different implant–prosthesis connection, either internal conical or internal hexagonal, used for the restoration of single crowns in the posterior mandible. The hypothesis was that implants with the internal conical connection lose less marginal bone than those with the internal hexagonal connection.

## 2. Materials and Methods

### 2.1. Study Design

This randomized clinical trial was designed following the CONSORT reporting guidelines. It was planned as a one-center study with allocation to either the control group (internal hexagonal-connection implants) or the test group (internal conical-connection implants). A sample size of 30 patients was planned at the beginning of the study.

Because the study was conducted at the Oral Surgery and Implant Dentistry Clinic of the School of Dentistry, University of Granada, the protocol was evaluated by the Institutional Ethics Committee for Research in Humans (University of Granada). It was approved and registered with number 213/CEIH/2016. In addition, the study protocol was registered at clinicaltrial.gov (NCT02975674). The protocol was developed in accordance with the Helsinki Declaration of the World Medical Association, the standard of clinical investigation of medical devices for human subjects (ISO 14155:2011) and the Directive regarding good clinical practices (2001/20/EC). Before any study procedure was initiated, each patient was informed about the study and asked to sign an informed consent form.

### 2.2. Participants

General inclusion criteria for conventional single implants were established for this study. Particularly, the patient must have been older than 18 years, healthy and with a missing single molar or premolar tooth in the presence of both adjacent and antagonist healthy teeth. We only included completely healed sites (more than 4 months after tooth extraction, Type 4 according to the 15th European Workshop of Periodontology on Bone Regeneration) [19]. Exclusion criteria included the need for bone or soft tissue augmentation, conditions that could modify healing or bone metabolism, smokers of more than 10 cigarettes/day and pregnant women. If any other dental disease was detected beside the missing tooth to be replaced, inclusion in the study was withheld until treatment of such condition.

### 2.3. Interventions

**Figure 1:**
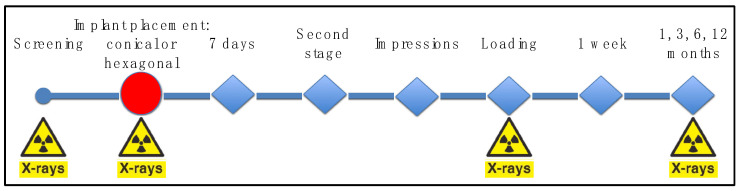
Diagram representing the study visits.

All the implants were placed by the same surgeon (P.G.-M.) assisted by the same PhD student (A.C.-J.). The study’s variables were registered by the same examiners (L.L.-C. and R.J.A.-R.). Implant placement followed a conventional technique after raising a full-thickness mucoperiosteal supracrestal flap. The implant site was drilled in the bone following the protocol and drilling sequence recommended by the company (Oxtein Iberia S.L., Zaragoza, Spain), which is the same for both types of implants: high-speed drilling (1200 rpm), profuse irrigation with sterile saline and a maximum of 55 Ncm torque. After the implant site was drilled, allocation to each study group was determined, so that an internal hexagonal connection (control; N35 implant, Oxtein Iberia S.L.) or an internal conical-connection implant (test; M12 implant, Oxtein Iberia S.L.) was inserted, always with a torque below 80 Ncm. The implant shoulder was always placed at the level of the buccal bone. The flap was carefully sutured with 4/0 surgical silk (Laboratorios Aragó, Barcelona, Spain). Eight weeks later, the second surgical phase was conducted to place a healing abutment. Dental impressions were taken 2 weeks later and metal ceramic screw-retained crowns were cast over pre-machined metal-base abutments. After approximately two weeks, each crown was installed over the implant. The prosthetic phase was conducted by the same operators (A.C.-J., E.S.-F. and M.P.-M.). At implant placement, prosthesis delivery and 1, 3, 6 and 12 months after implant loading, periapical radiographs of the area were obtained. A diagram representing the study sequence of visits and procedures is presented in Figure 1.

### 2.4. Outcomes

**Figure 2:**
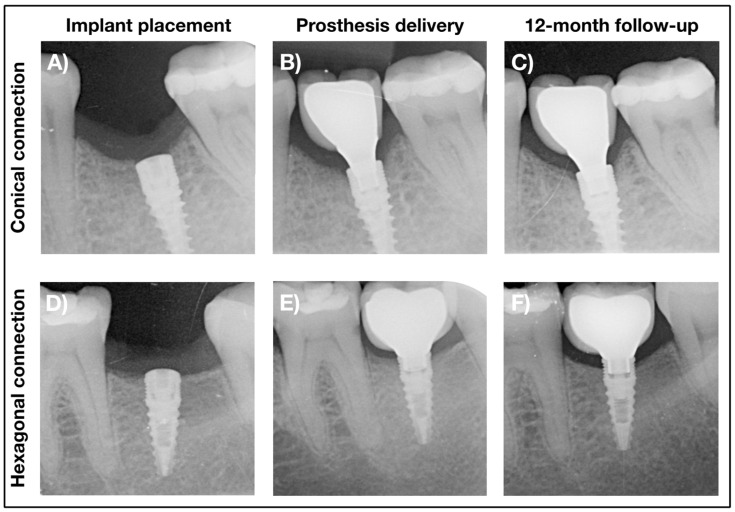
Representative radiographs of the implants at implant placement (**A**,**D**), prosthesis delivery (**B**,**E**) and 12-month follow-up (**C**,**F**) for the conical (**A**–**C**) and hexagonal (**D**–**F**) connection groups.

The primary outcome measure of this study was marginal bone level (MBL) change from prosthesis delivery to the 12-month follow-up (Figure 2). Other MBL measurements were obtained at different follow-up visits, both at the implants, prosthetic restoration and adjacent teeth. All MBL measures of the implants took the implant shoulder as reference; for teeth, the cement–enamel junction was used as the reference point. Linear measurements were conducted by an experienced dentist specializing in dental implantology (M.P.-M.) using the Image J software (National Institutes of Health, Bethesda, MD, USA). Each image was internally calibrated considering the known dimensions of the implant. The radiographs of the implant area were obtained by parallel technique with an X-ray positioner and scanned to a computer in order to conduct the measurements.

Several clinical measurements were also recorded at the time of implant placement: occlusal height, buccolingual width (before and after raising the flap), mesiodistal distance, width of the keratinized mucosa, vertical soft tissue thickness and thickness of the buccal plate after implant placement. The latter was also registered during dental impressions and when the prosthesis was delivered. The width of the keratinized mucosa as well as the papilla index (0 = no papilla; 1 ≤ 50% filling of the interproximal area; 2 ≥ 50% filling; 3 = ideal papilla; 4 = overgrowth) [20] were also registered at each follow-up visit.

### 2.5. Sample Size and Statistical Power

The study was originally designed as a pilot study. Thus, the power achieved with this study was evaluated with a post hoc test taking into consideration the means and the standard deviations of the main outcome measure of the study for those patients evaluated at the 12-month follow-up visit. G*Power 3.1.9.3 for Mac OS was used.

### 2.6. Randomization

A clinic staff member not involved in the clinical trial used the Qminim software to randomize the allocation of each individual while balancing the groups in terms of gender, location, and type of bone.

### 2.7. Blinding

Because of the macroscopic characteristics of the implant–prosthesis connection, only the patient and the clinical examiners (L.L.-C. and R.J.A.-R.) could be blinded with regard to the group assignment. Neither the surgeon (P.G.-M.), the restorative dentist (A.C.-J.) nor the data analyst (M.P.-M.) were blinded.

### 2.8. Statistical Analysis

For categorical data, percentages were calculated and tested with the chi-squared test. Means and standard deviations were calculated for continuous variables. Because of the sample size and data distribution, statistical differences between groups in continuous outcome measures were analyzed by means of the nonparametric independent samples Mann–Whitney U test. To explore the possible correlation of any other variable in the main outcome measures, Spearman’s rho correlation coefficient was analyzed as well. Prism 7 for Mac OS X (version 7.0a) (Graphpad Software Inc., San Diego, CA, USA) was used for creating the graphs representing the data. Statistical analyses were conducted using IBM SPSS Statistics 26 (release 26.0.0.2) (IBM Corporation, Armond, NY, USA). In all cases, *p* < 0.05 was set as the limit for statistical significance.

## 3. Results

**Figure 3:**
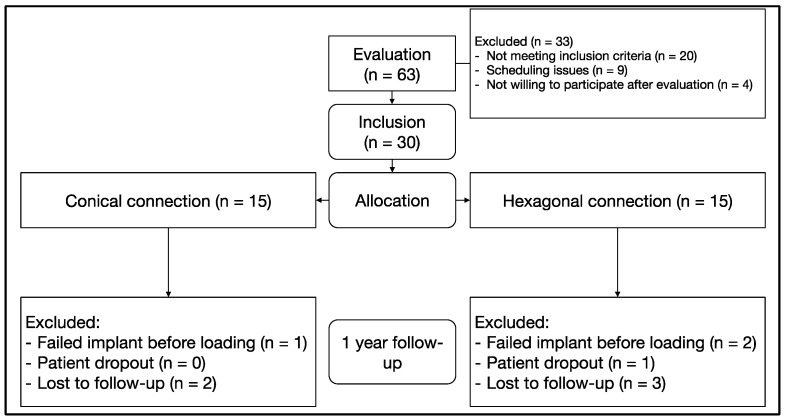
CONSORT diagram.

A total of 63 patients were screened for participation in the study between March 2017 and July 2019. A total of 30 patients were included in the study and randomized to the test and control groups (n = 15 patients per group). Mainly because of the COVID-19 pandemic, not all the patients completed the 12-month follow-up and/or intermediate visits. This information is summarized in Figure 3. With the data obtained with the patients included in the final evaluation setting the α-error at 0.05, the 1-β error (power) was 0.918.

**Figure 4:**
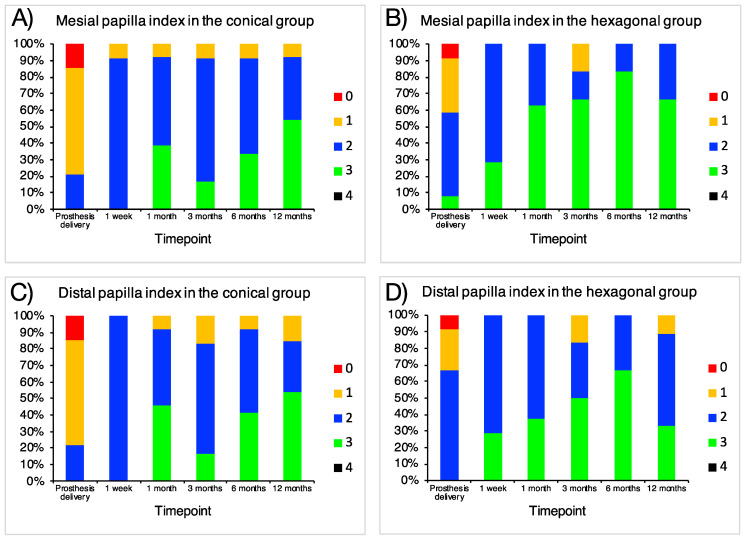
Percentages of each papilla index at the different timepoints at (**A**,**B**) mesial and (**C**,**D**) distal sites of (**A**,**C**) conical and (**B**,**D**) hexagonal-connection implants; 0 = no papilla; 1 ≤ 50% filling of the interproximal area; 2 ≥ 50% filling; 3 = ideal papilla; 4 = overgrowth.

Table 1 summarizes demographic and clinical data. The average age of the included patients was 43 (22, 60) and 46 (21, 71) (*p* = 0.589, independent samples Mann–Whitney U test) for the conical and hexagonal connection groups, respectively. Eight and nine patients, respectively, were females in the conical and hexagonal connection groups (*p* = 0.713, chi-squared test). Around 80% in each group were non-smokers and 93.3% did not consume alcohol. No systemic disease was reported by any patient. Except for one case of vertical fracture, the reason for tooth extraction in all the remaining cases was extensive caries. None of the evaluated clinical parameters regarding the implant or the surrounding area showed statistical differences between the groups except for the occlusal height (8.60 (1.35) vs. 7.07 (1.87); conical vs. hexagonal group, respectively; *p* = 0.023, independent samples Mann–Whitney U test) (Table 1). In all the cases, appropriate esthetic results were achieved, with no statistically significant differences, as represented by the papilla index (Table 1 and Figure 4).

At the second stage, a total of two implants in the hexagonal connection group and one in the conical connection group were not osseointegrated. All the other implants were restored according to the proposed protocol. During the course of 1 year of follow-up, one case suffered ceramic chipping that was restored, one case presented suppuration at 8 months due to food impaction that was solved by unscrewing the crown and cleaning and two more cases suffered from crown loosening (one after 1 month of loading and one after 3 months). All of these complications occurred in the hexagonal connection group.

**Figure 5:**
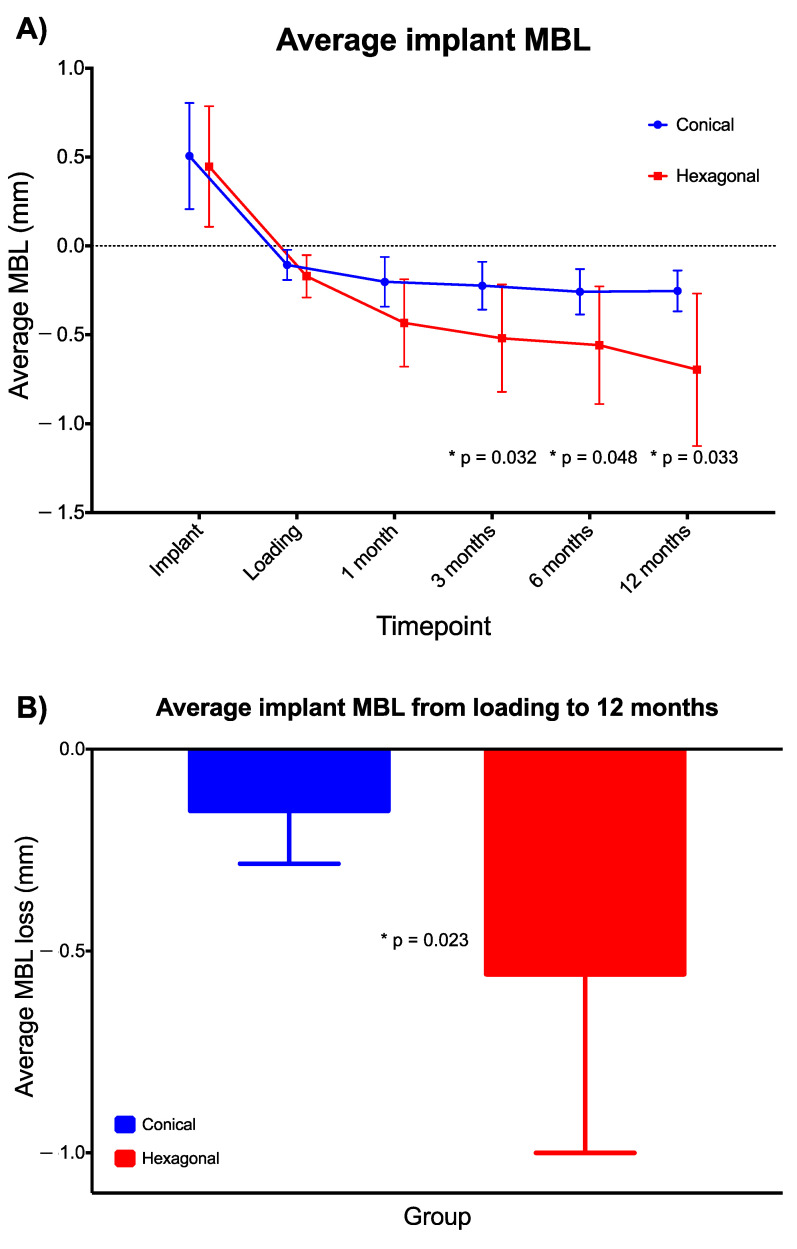
(**A**) Representation of the average implant MBL over time. (**B**) Average implant MBL change from prosthesis delivery to the 12-month follow-up. * Independent samples Mann–Whitney U test. Error bars represent SD.

In terms of radiographical data (Table 2), although no difference between groups was found in the average MBL at prosthesis delivery (−0.11 (0.08) vs. −0.17 (0.12); conical vs. hexagonal; *p* = 0.176, independent samples Mann–Whitney U test), significant differences were observed at the 3- (−0.22 (0.13) vs. −0.52 (0.30); conical vs. hexagonal), 6- (−0.26 (0.13) vs. −0.56 (0.33); conical vs. hexagonal) and 12-month (−0.25 (0.12) vs. −0.70 (0.43); conical vs. hexagonal) follow-up visits (*p* = 0.032, *p* = 0.048 and *p* = 0.033, respectively, independent samples Mann–Whitney U test) (Figure 5A). In addition, the average MBL change from prosthesis delivery to the 12-month follow-up was also significantly different between groups (−0.15 (0.13) vs. −0.56 (0.44); conical vs. hexagonal; *p* = 0.023, independent samples Mann–Whitney U test) (Figure 5B).

**Figure 6:**
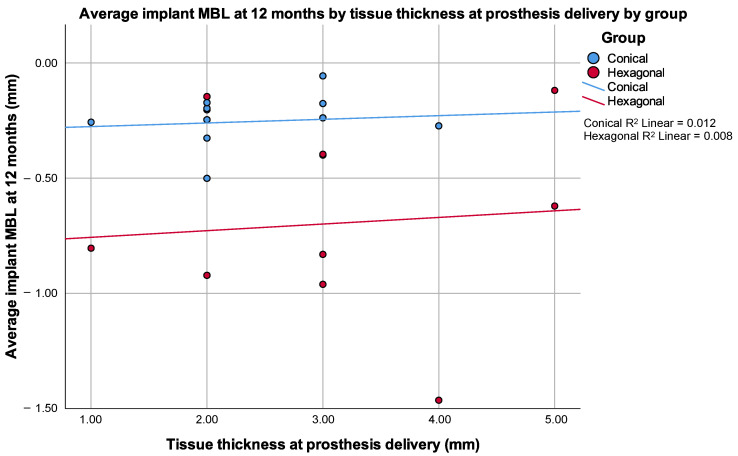
Scattered plot by group of the average implant MBL at 12 months by tissue thickness at prosthesis delivery. Note that Spearman’s rho correlation coefficient was 0.088 (*p* = 0.787) for the conical group and 0.120 (*p* = 0.759) for the hexagonal group.

In addition to the absence of differences between groups in any other radiographic measurements, correlations between radiographic measurements on the adjacent teeth, buccal bone to implant, tissue thickness or keratinized tissue and radiographic measurements were not consistent at the different timepoints. Particularly, no statistically significant correlation was found between tissue thickness at prosthesis delivery and the average MBL at any timepoint neither globally nor when analyzed independently by group (Figure 6).

## 4. Discussion

This study analyzed differences in the marginal bone level (MBL) around implants with similar macroarchitecture and surface properties. Two groups were established depending on the implant–prothesis connection: internal conical or internal hexagonal. In all the cases, single implants were placed and restored with single crown restorations. Statistical differences between both types of connections are appreciable in the final MBL measured after 12 months of prosthetic loading. However, other important variables under analysis, including (1) width of the keratinized tissue, (2) soft tissue thickness, (3) distance from the buccal plate to the implant shoulder, (4) buccolingual bone availability and (5) implant length or diameter, did not show any influence on the final MBL. Our findings contradict several studies previously published in the scientific literature [21,22,23,24]. We must mention though that most of our implants were above the red lines established for those important variables: (1) more than 2 mm of keratinized tissue, (2) more than 2 mm of tissue thickness and (3) more than 1 mm of buccal bone to implant (Table 1 and Figure 6). In any case, the influence of these parameters is being questioned in more recent studies, particularly that related to the thickness of the tissue [25,26,27].

There are few in vivo studies in humans comparing the impact of both types of connection in MBL. A recent meta-analysis suggested that a tapered connection offers significantly less MBL compared with a non-tapered connection [16]. However, except the study by Cannata et al. [17], the remaining six studies included in Yu’s meta-analysis compared different implant typologies, with different implant surfaces and macrostructure: Astra Osseospeed implant vs. Certain Prevail implant [28,29]; Ankylos vs. Certain Prevail [30,31]; Nobel Active vs. Nobel Replace [32,33]. Thus, conclusions from these studies must be compared with caution to those found in this study.

Clinical studies using the same comparison as here (internal conical vs. internal hexagonal connection) are really scarce. In fact, we only identified two studies. Szyszkowski and Kozakiewicz reported significantly lower average marginal bone loss in conical-connection implants compared with internal hexagonal-connection implants at all of their follow-up visits: 0.68 ± 0.59 mm vs. 0.99 ± 0.89 mm (12 months), 0.78 ± 0.80 mm vs. 1.12 ± 1.00 mm (24 months), 0.83 ± 0.87 mm vs. 1.22 ± 1.03 mm (36 months) and 0.96 ± 1.02 mm vs. 1.30 ± 1.15 mm (60 months) [18]. However, this was a retrospective study conducted in implants from different companies; thus, those implants had different micro- and macrocharacteristics. In addition, there was an important discrepancy in the number of implants included in each group: 480 internal hexagonal- vs. 60 internal conical-connection implants. Moreover, in contrast with our study, where all implants were located in the posterior inferior maxilla, their implants were placed in different locations of the maxilla and mandible. The other study we found comparing internal conical- vs. internal hexagonal-connection is the only controlled randomized prospective multicenter study available in the literature using implants with similar micro- and macrodesign and from the same manufacturer. The authors were unable to find statistically significant differences between conical- and hexagonal-connection implants after one year of follow-up: 0.60 ± 0.62 mm vs. 0.56 ± 0.53 mm, respectively [17]. Our results showed more bone preservation around the internal conical-connection implants between the time of prosthesis delivery and the one-year follow-up visit: from −0.11 ± 0.08 mm to −0.25 ± 0.12 mm in the conical connection group vs. −0.17 ± 0.12 mm to −0.70 ± 0.43 mm in the hexagonal connection group. In fact, the internal conical-connection implants seemed to show a trend of bone stability between the biological width establishment and the subsequent follow-up visits. Similar findings were recently reported, both in magnitude and pattern [34]. In turn, the internal hexagonal-connection implants showed a slow but continuous decrease in the level of bone over time (Table 2 and Figure 5A).

This study corroborates previous findings that relate MBL with the biological width establishment in a nonlinear progression [1]. We found statistical differences between groups after only 3 months from prosthesis delivery. These differences were present up to the final follow-up visit in our study. Before the 3-month visit, no differences were observed. Thus, we may say that before the final maturation of the supracrestal soft tissue around the implant–prosthesis complex, the connection has no influence. When the whole period is observed, marginal bone loss was higher (−0.56 ± 0.44 mm vs. −0.15 ± 0.13 mm from prosthesis delivery to 1 year of follow-up) and started earlier (−0.43 ± 0.25 mm vs. −0.20 ± 0.14 mm one month post-loading) in the internal hexagonal- than in the internal conical-connection implants, respectively. Despite the apparently low clinical impact of these magnitudes of marginal bone loss, we have to keep in mind that, as demonstrated by our group in earlier studies [1], those implants that lose more than 0.44 mm before 6 months after loading (defined as high bone loser type) would be at higher risk of losing more than 2 mm after 18 months. Thus, early bone loss, although not clinically relevant at the precise moment of the evaluation, might be a good indicator of the mid- and long-term prognosis. Then, clinical strategies for closer follow-up and care could be implemented in those cases in order to prevent future complications.

There are different explanations that could justify these differences:

A. Presence of microorganisms.

It has been argued that the presence of bacteria in the peri-implant sulcular environment conditions MBL. In fact, Piattelli et al. established that the closer the crown is to the bone, the larger the bone resorption. This occurs as a result of an inflammation zone initiated by the presence of bacteria in the implant–prosthesis interface [35]. This idea has been greatly reinforced with the introduction of the platform switching concept. It aims at distancing the bacterial reservoir from the bone as far as possible because in real clinical settings, all kinds of prosthetic connections suffer some level of bacterial contamination [36]. In fact, systematic reviews of in vivo studies show that there is no connection capable of totally avoiding bacterial contamination [13], although this can be achieved in highly controlled in vitro environments [37]. For example, D’Ercole et al., in an in vitro study comparing internal hexagonal vs. conical implant–abutment connections found lower infiltration rates in the internal conical-connection implants. However, the differences were not significant [38]. In any case, standardized clinical studies are needed to differentiate the microbiota present in both types of connections.

B. Load distribution from the implant to the bone.

Several finite elements analysis studies explained how the occlusal load could be distributed in the cortical area of the bone and its impact on bone marginal loss [39]. In fact, different solutions were introduced in implants, such as microthreads [40,41], to decrease or eliminate this tension. It is logical to understand that the distribution of load energy from the crown to the implant or from the implant to the bone would not be equal in the different types of connections and it would also depend on the microarchitecture of the bone surrounding the implant. Thus, the crown–implant–bone occlusal load distribution system is key. On the contrary, Hung et al. ensured that implants with an internal hexagonal connection show higher compressive strength than those with an internal hexagonal connection in combination with the Morse taper design [42]. It must be kept in mind though that the strength of a connection does not necessarily mean that the microbial contamination would be less or the load distribution better.

C. Micromovements between the prosthesis and the implant.

The micromovements between prosthetic components and implants have been classically defined as one of the main causes of MBL, either (1) by allowing the contamination of bacteria into the gaps created in the interface, (2) by establishing pumping or flow phenomena of microorganisms [43], (3) by promoting material wear and release of debris to the local environment [44] or (4) by deteriorating the mechanical properties of the elements in the interface [45]. Zipprich et al. demonstrated a reduction in the formation of microgaps and micromovements in implants with an internal conical connection compared to implants with internal flat connections not only in static loading, but also in dynamic lateral loading [46]. This is also true if angled abutments are used in internal conical connections [47], although this comparison was made to external hexagonal connections.

We must also consider the method for fabricating the prosthesis. In our study, all the cases were restored with screw-retained metal ceramic single crowns over UCLA abutments with pre-machined metal bases. Prosthesis retention has been the focus of numerous studies. None of the retention methods is free of potential complications [48], including the newly proposed microlocking systems [49]. The screw-retained method seems, however, to be the most predictable and less problematic in terms of biological complications [50]. In turn, the screw-retained method suffers from more frequent mechanical complications, including unscrewing of the prosthesis, which can damage the connection and result in higher bone loss [51]. This study found some of these complications, but always in the hexagonal connection group. Additionally, as known from different studies, new technologies such as CAD/CAM and posterior milling or laser-sintered processing may offer better results in terms of adjustment of the prothesis and the implant [52,53,54]. In addition, milling and sintering offer smoother surfaces that would retain less microorganisms and induce fewer inflammatory reactions [55]. However, the castable method is still the most common one, which is the reason why we used this method in our study.

In other aspects, a recent meta-analysis suggested that internal hexagonal-connection implants provide better esthetic results in terms of the pink esthetic score/white esthetic score (PES/WES) [56]. Although in absolute values it may seem that we found similar results, we were unable to find any statistical difference between our groups in the papilla index (Table 1 and Figure 4).

Beside our findings, one of the main strengths in our design is, as in Cannata’s study [17], the use of implants with similar macrogeometry and surface characteristics. Furthermore, all of the implants were placed in inferior posterior sites. However, there are also some limitations. The study was designed as a pilot study, so the sample size was initially reduced. The recruitment was slow due to the inclusion criteria requiring healthy adjacent and opposite teeth. The percentage of implant failures might seem high but we have to consider the limited sample size. In addition, and unfortunately, this study had many final visits planned for 2020, when the COVID-19 pandemic occurred, which is why some patients declined to conclude the final follow-up, and others could not travel to the study site. Altogether, we must be aware of the circumstances and recognize that our results should be considered with caution. In any case, our results show statistical power, as described in the corresponding section. We believe that valuable information can be extracted from our results in this insufficiently reported topic. Our results confirm the potency of the connection variable in the MBL outcome.

## 5. Conclusions

According to our initial hypothesis, in patients restored with single-unit implants in the posterior mandible, internal conical-connection implants show less marginal bone loss after 12 months of follow-up from prosthesis delivery than internal hexagonal-connection implants. In this clinical study, no other clinical parameter was relevant in the development and progression of marginal bone loss around implants. Further studies are needed to elucidate deeper knowledge about the role of the different type of connections in MBL.

## Figures and Tables

**Table 1:** Description and comparison of demographic and clinical variables.

	Test Group (Conical Connection) n = 15 (50.0%)				Control Group (Hexagonal Connection) n = 15 (50.0%)				*p*-Value *
**Age** (mean (min, max)) (years)	45 (21, 71)								
	43 (22, 60)				46 (21, 71)				0.589
**Gender** (n (% within the group))									0.713
Female	8 (53.3)				9 (60.0)				
Male	7 (46.7)				6 (40.0)				
**Smoking** (n (%))									0.624
No	13 (86.7)				12 (80.0)				
Low (<5 cigarettes/day)	2 (13.3)				3 (20.0)				
**Alcohol** (n (%))									1.000
No	14 (93.3)				14 (93.3)				
Low (<10 g/day)	1 (6.7)				1 (6.7)				
**Mesiodistal distance** (mean (SD)) (mm)	10.00 (3.30)				10.07 (1.49)				0.832
**Occlusal height** (mean (SD)) (mm)	8.60 (1.35)				7.07 (1.87)				0.023
**Buccolingual width** (mean (SD)) (mm)									
Before flap raising	7.47 (1.41)				8.00 (2.45)				0.933
After flap raising	7.13 (1.51)				7.53 (1.92)				0.898
**Implant diameter** (n (%))									0.666
**3.5 mm**	4 (26.7)				3 (20.0)				
**4.0 mm**	11 (73.3)				12 (80.0)				
**Implant length** (n (%))									0.464
**10.0 mm**	6 (40.0)				8 (53.3)				
**11.5 mm**	9 (60.0)				7 (46.7)				
**Buccal bone to implant** (mean (SD)) (mm)	1.57 (0.86)				1.53 (0.83)				1.000
**Tissue thickness** (mean (SD)) (mm)									
Implant placement	1.93 (0.59)				2.70 (1.31)				0.077
Impressions	2.50 (0.76)				2.62 (1.04)				0.758
Prosthesis delivery	2.43 (0.76)				2.92 (1.24)				0.338
**Width of the keratinized tissue** (mean (SD)) (mm)									
Implant placement	3.27 (1.16)				2.93 (1.34)				0.541
Prosthesis delivery	2.71 (0.83)				2.42 (1.00)				0.442
1 week	2.67 (0.65)				2.14 (0.38)				0.073
1 month	2.54 (0.66)				2.13 (0.35)				0.125
3 months	2.42 (0.79)				2.33 (0.82)				1.000
6 months	2.42 (0.51)				2.17 (0.75)				0.522
12 months	2.23 (0.73)				2.44 (0.73)				0.488
**Papilla index** (% within a visit) (mesial) **	0	1	2	3	0	1	2	3	
Prosthesis delivery	14.3	64.3	21.4	0.0	8.3	33.3	50.0	8.3	0.248
1 week	0.0	8.3	91.7	0.0	0.0	0.0	71.4	28.6	0.121
1 month	0.0	7.7	53.8	38.5	0.0	0.0	37.5	62.5	0.474
3 months	0.0	8.3	75.0	16.7	0.0	16.7	16.7	66.7	0.058
6 months	0.0	8.3	58.3	33.3	0.0	0.0	83.3	83.3	0.131
12 months	0.0	7.7	38.5	53.8	0.0	0.0	33.3	66.7	0.645
**Papilla index** (% within a visit) (distal) **	0	1	2	3	0	1	2	3	
Prosthesis delivery	14.3	64.3	21.4	0.0	8.3	25.0	66.7	0.0	0.064
1 week	0.0	0.0	100.0	0.0	0.0	0.0	71.4	28.6	0.050
1 month	0.0	7.6	46.2	46.2	0.0	0.0	62.5	37.5	0.621
3 months	0.0	16.7	66.7	16.7	0.0	16.7	33.3	50.0	0.301
6 months	0.0	8.3	50.0	41.7	0.0	0.0	33.3	66.7	0.535
12 months	0.0	15.4	30.8	53.8	0.0	11.1	55.6	33.3	0.506

Note: * *p*-value: independent samples Mann–Whitney U test for continuous variables and chi-squared test for categorical variables; ** there were no cases with the papilla index higher than 3.

**Table 2:** Description and comparison of radiographical variables (in mm except for the crown-to-implant ratio).

	Test Group (Conical Connection)		Control Group (Hexagonal Connection)		*p*-Value *
	Mean (SD)	Median	Mean (SD)	Median	
**Distance from the implant to the anterior tooth**	4.94 (1.55)	5.47	4.22 (1.09)	4.29	0.118
**Distance from the implant to the posterior tooth**	4.22 (1.51)	3.99	3.98 (1.03)	4.11	0.683
**Crown length**	10.84 (1.28)	10.68	10.18 (2.39)	9.53	0.085
**Crown-to-implant ratio**	1.00 (0.11)	1.04	0.93 (0.19)	0.92	0.131
**Implant MBL** (mesial)					
Implant placement	0.47 (0.30)	0.45	0.45 (0.32)	0.34	0.806
Prosthesis delivery	−0.10 (0.09)	−0.11	−0.21 (0.16)	−0.17	0.037
1 month	−0.20 (0.15)	−0.14	−0.38 (0.21)	−0.39	0.141
3 months	−0.21 (0.13)	−0.19	−0.48 (0.31)	−0.41	0.067
6 months	−0.24 (0.13)	−0.25	−0.52 (0.31)	−0.49	0.078
12 months	−0.23 (0.15)	−0.24	−0.60 (0.41)	−0.75	0.058
**Implant MBL** (distal)					
Implant placement	0.54 (0.40)	0.43	0.44 (0.41)	0.41	0.389
Prosthesis delivery	−0.11 (0.10)	−0.10	−0.13 (0.09)	−0.13	0.520
1 month	−0.21 (0.16)	−0.23	−0.49 (0.31)	−0.39	0.099
3 months	−0.24 (0.15)	−0.23	−0.56 (0.36)	−0.60	0.103
6 months	−0.28 (0.14)	−0.25	−0.60 (0.43)	−0.62	0.256
12 months	−0.28 (0.15)	−0.29	−0.79 (0.48)	−0.85	0.018
**Average implant MBL**					
Implant placement	0.51 (0.30)	0.46	0.45 (0.34)	0.40	0.461
Prosthesis delivery	−0.11 (0.08)	−0.12	−0.17 (0.12)	−0.17	0.176
1 month	−0.20 (0.14)	−0.18	−0.43 (0.25)	−0.36	0.129
3 months	−0.22 (0.13)	−0.20	−0.52 (0.30)	−0.53	0.032
6 months	−0.26 (0.13)	−0.25	−0.56 (0.33)	−0.51	0.048
12 months	−0.25 (0.12)	−0.24	−0.70 (0.43)	−0.80	0.033
**MBL change from implant placement to prosthesis delivery**					
Mesial	−0.55 (0.34)	−0.56	−0.73 (0.40)	−0.56	0.274
Distal	−0.58 (0.28)	−0.48	−0.65 (0.48)	−0.58	0.980
Average	−0.56 (0.26)	−0.51	−0.69 (0.42)	−0.62	0.520
**MBL change from prosthesis delivery to 12 months**					
Mesial	−0.13 (0.17)	−0.14	−0.43 (0.41)	−0.53	0.069
Distal	−0.17 (0.17)	−0.14	−0.68 (0.51)	−0.72	0.018
Average	−0.15 (0.13)	−0.13	−0.56 (0.44)	−0.64	0.023

Note: * *p*-value: independent samples Mann–Whitney U test.

## Data Availability

Data supporting the study findings are available from the corresponding author upon request.
